# Role of *GSK-3β* in the Osteogenic Differentiation of Palatal Mesenchyme

**DOI:** 10.1371/journal.pone.0025847

**Published:** 2011-10-14

**Authors:** Emily R. Nelson, Benjamin Levi, Michael Sorkin, Aaron W. James, Karen J. Liu, Natalina Quarto, Michael T. Longaker

**Affiliations:** 1 Hagey Laboratory for Pediatric Regenerative Medicine, Plastic and Reconstructive Surgery Division, Department of Surgery, Stanford University School of Medicine, Stanford, California, United States of America; 2 Department of Craniofacial Development, King's College London, London, United Kingdom; 3 Dipartimento di Scienze Chirurgiche, Anestesiologiche-Rianimatorie e dell ‘Emergenza “Giuseppe Zannini,” Universita’ degli Studi di Napoli Federico II, Napoli, Italy; 4 Institute for Stem Cell Biology and Regenerative Medicine, Stanford University, Stanford, California, United States of America; University of Massachusetts Medical, United States of America

## Abstract

**Introduction:**

The function of *Glycogen Synthase Kinases 3β* (*GSK-3β*) has previously been shown to be necessary for normal secondary palate development. Using *GSK-3ß* null mouse embryos, we examine the potential coordinate roles of *Wnt* and *Hedgehog* signaling on palatal ossification.

**Methods:**

Palates were harvested from *GSK-3β*, embryonic days 15.0–18.5 (e15.0–e18.5), and e15.5 *Indian Hedgehog* (*Ihh*) null embryos, and their wild-type littermates. The phenotype of *GSK-3β* null embryos was analyzed with skeletal whole mount and pentachrome stains. Spatiotemporal regulation of osteogenic gene expression, in addition to *Wnt* and *Hedgehog* signaling activity, were examined *in vivo* on *GSK-3β* and *Ihh* +/+ and −/− e15.5 embryos using *in situ* hybridization and immunohistochemistry. To corroborate these results, expression of the same molecular targets were assessed by qRT-PCR of e15.5 palates, or e13.5 palate cultures treated with both *Wnt* and *Hedgehog* agonists and anatagonists.

**Results:**

*GSK-3β* null embryos displayed a 48 percent decrease (*p<0.05) in palatine bone formation compared to wild-type littermates. *GSK-3β* null embryos also exhibited decreased osteogenic gene expression that was associated with increased *Wnt* and decreased *Hedgehog* signaling. e13.5 palate culture studies demonstrated that *Wnt* signaling negatively regulates both osteogenic gene expression and *Hedgehog* signaling activity, while inhibition of *Wnt* signaling augments both osteogenic gene expression and *Hedgehog* signaling activity. In addition, no differences in *Wnt* signaling activity were noted in *Ihh* null embryos, suggesting that canonical *Wnt* may be upstream of *Hedgehog* in secondary palate development. Lastly, we found that *GSK-3β* −/− palate cultures were “rescued” with the *Wnt* inhibitor, Dkk-1.

**Conclusions:**

Here, we identify a critical role for *GSK-3β* in palatogenesis through its direct regulation of canonical *Wnt* signaling. These findings shed light on critical developmental pathways involved in palatogenesis and may lead to novel molecular targets to prevent cleft palate formation.

## Introduction

Craniofacial birth defects, such as cleft lip and/or palate, can cause extensive physical and psychosocial complications, representing a significant socioeconomic burden. Multiple operations are often required to surgically reconstruct the palate defect, and affected children continue to suffer from complications with speech, feeding, dentition, hearing, and other craniofacial growth deficiencies. Although several genes have been associated with a small percentage of cleft palate defects, the etiology of the majority of cases remains elusive.

The function of *Glycogen Synthases Kinase-3β* (*GSK-3β*) has previously been shown to be critical for normal craniofacial development [1]. GSK-3ß is a key integrator of various molecular pathways and thus functions as an essential component for normal development. For instance, deletion of both *GSK-3* isoforms (designated *α* and *β*), which differ primarily in their N-terminal domains, results in early embryonic lethality [2]. As a kinase, GSK-3ß acts to inhibit rather than activate its substrate. Therefore, inactivation of GSK-3ß typically results in the release of its inhibition on the specific pathways it regulates, resulting in an upregulation of signaling activity in these pathways. GSK-3ß is an important regulator of a number of molecular pathways, such as *Wnt*, *Hedgehog*, NFAT, and insulin signaling [1]. Of these pathways, the precise regulation of both *Wnt* and *Hedgehog* signaling may be necessary for normal craniofacial development, as disruptions in either of these pathways can lead to craniofacial abnormalities, including palatal clefting [3], [4], [5].

In mice, secondary palate development begins on embryonic day 12.5 (e12.5). At this time, the maxillary processes give rise to bilateral palatal shelves, consisting of a mesodermal core that is surrounded by neural crest cells in addition to pharyngeal endoderm and ectoderm [6]. By e15.5, palatal fusion is complete and mesenchymal condensation occurs, followed by the osteogenic differentiation of the palatal mesenchyme, leading to the formation of the palatine bone in the secondary palate [6]. It has been shown that both *Wnt* and *Hedgehog* pathways play central roles in the differentiation of mesenchymal progenitor cells down an osteoblastic lineage [7], [8], [9], [10], [11]. More importantly, both higher levels of *Wnt* and lower levels of *Hedgehog* signaling have been shown to inhibit osteogenic differentiation of mesenchymal cells [11], [12], [13], [14], indicating that pathways capable of inhibiting *Wnt* (such as *GSK3ß*) and stimulating *Hedgehog* may play an important role in bone formation.

In light of the fact that both *Wnt* and *Hedgehog* signaling have previously been shown to be regulated by GSK-3ß, and implicated in normal craniofacial development and mesenchymal osteogenesis, we set out to examine the potential coordinate roles of *Wnt* and *Hedgehog* signaling on the ossification program of the palatine bones in the developing secondary palate. More specifically, we hypothesize that the loss of *GSK-3ß* leads to a diminutive palatine bone and impaired palatal ossification due to alterations in both *Wnt* and *Hedgehog* signaling. Moreover, we hypothesize that the inactivation of *GSK-3β* leads to enhanced *Wnt* signaling, which subsequently leads to decreases in *Hedgehog* signaling, and that it is in this setting of enhanced *Wnt* and decreased *Hedgehog* signaling that palatal ossification is compromised.

## Results

### *GSK-3β* Null Embryos Display Diminutive Palatine Bones in the Developing Secondary Palate

**Figure 1:**
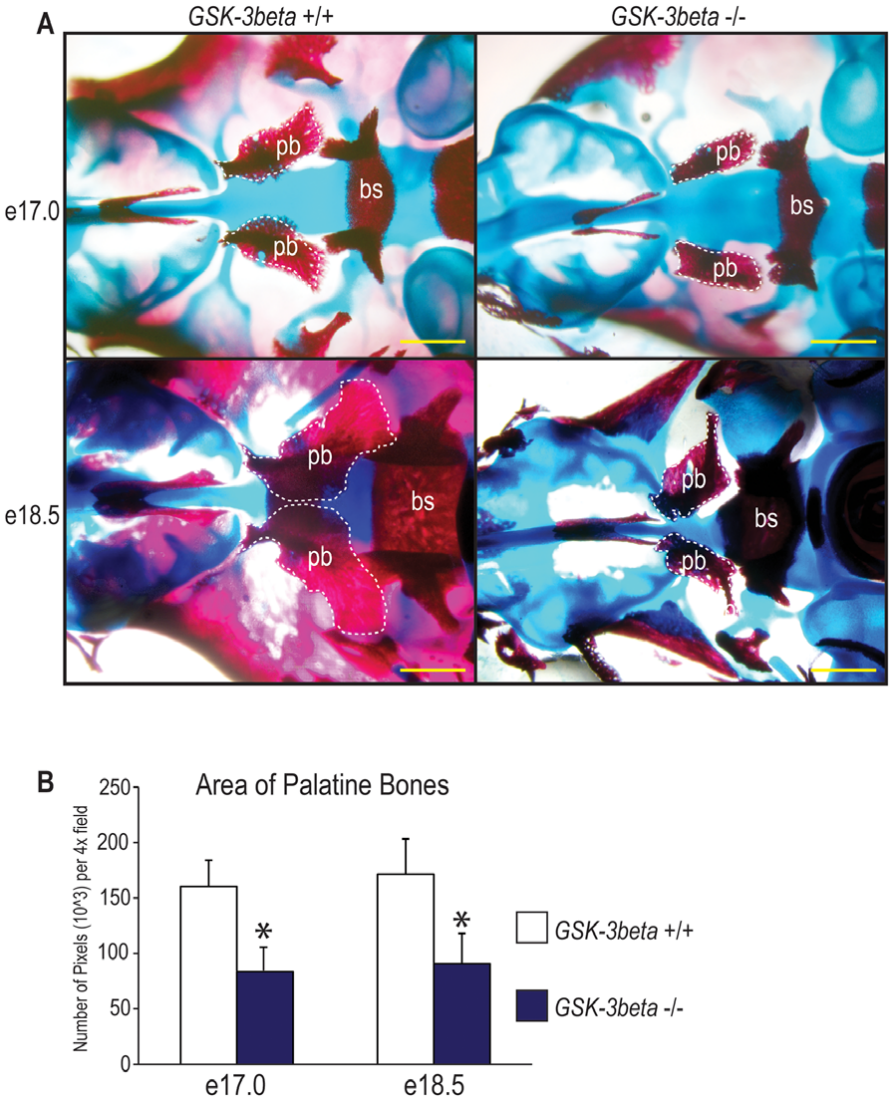
*GSK-3β* −/− palatal phenotype. (A) Bone and cartilage whole mount staining of *GSK-3β* +/+ (left) and *GSK-3β* −/− (right) embryos at e17.0 (first row) and e18.5 (second row). In this stain, bone appears red and cartilage appears blue. White dashed lines indicate the location of the palatine bones (pb), for emphasis. The basisphenoid bone (bs) is labeled for orientation purposes. Scale bars represent 600 µm. (B) The area of the palatine bones was quantified at both e17.0 and e18.5 using Adobe Photoshop, as the number of pixels per 4× field. The area of the palatine bones displayed a 48 percent decrease in the *GSK-3β* null embryos at both e17.0 and e18.5. N = 3, *p<0.05.

We have previously shown that *GSK-3β* null embryos display complete clefting of the secondary palate. In order to more thoroughly analyze the *GSK-3β* null embryo palatal phenotype, we performed a whole mount skeletal stain looking specifically at the palatine bones (**Figure 1**), as the palatine processes of the maxilla and the horizontal lamina of the palatine bones form the secondary palate, which is clefted in the *GSK-3β* null embryo. Based on alizarin red staining of the secondary palate, the palatine bones are appreciably smaller in the *GSK-3β* null embryos when compared to controls (**Figure 1A**). Next, we quantified the area of the palatine bones, which were significantly decreased in the *GSK-3β* −/− embryos when compared to controls at both e17.0 and e18.5 (**Figure 1B**). More specifically, when compared to wild-type littermates, the area of the palatine bones displayed a 48 percent decrease in the *GSK-3β* null embryos at both e17.0 and e18.5 (**Figure 1B**). These data clearly show that the area of the palatine bones in the developing palate of the *GSK-3β* null embryo remain consistently and significantly smaller in size than their wild-type littermates.

### *GSK-3β* Null Embryos Exhibit Inhibited Ossification of the Palatine Bones in the Developing Secondary Palate

**Figure 2:**
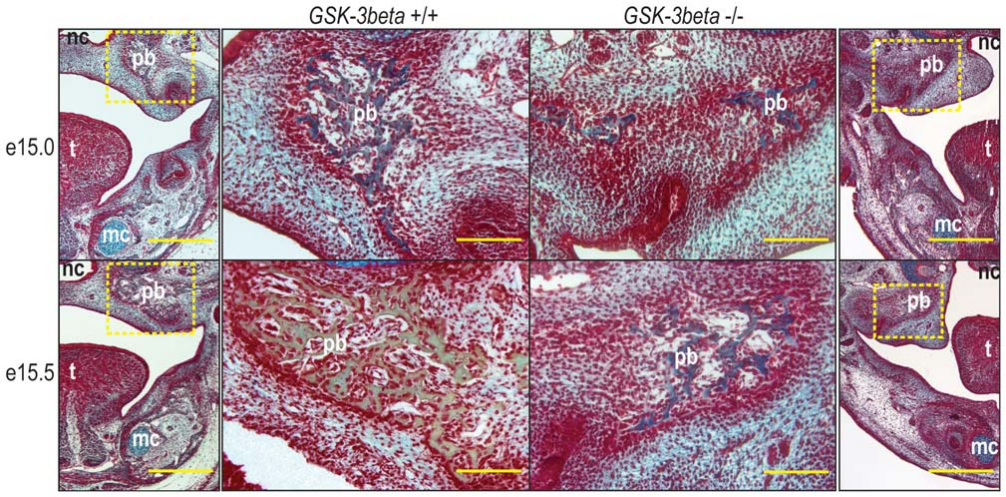
*GSK-3β* null embryos exhibit inhibited ossification of the palatine bone. Pentachrome staining of e15.0 (first row) and e15.5 (second row) coronal sections from *GSK-3β* +/+ (left) and *GSK-3β* −/− (right) embryos. In this stain, bone appears yellow. For orientation purposes, a low magnification image is shown in the outermost columns. A yellow box is drawn around the area seen in higher magnification in the center columns. (First row) At e15.0, *GSK-3β* +/+ embryos display trace amounts of bone (yellow staining) in the developing palatine bone (pb), while no bone is noted in *GSK-3β* −/− embryos. (Second row) At e15.5, the developing palatine bone of *GSK-3β* +/+ embryos is composed mostly of bone (yellow staining), while no bone is appreciated in *GSK-3β* −/− embryos. Tongue (t), Nasal Cavity (nc), and Meckel's Cartilage (mc) are labeled for orientation purposes. Scale bars represent 800 µm in the low magnification, outermost columns, and 100 µm in the high magnification, center columns.

Next, we analyzed the histology of the developing palate in order to confirm that *GSK-3β* null embryos exhibit inhibited ossification of the palatine bones. Since palatine bone formation begins at approximately e14.5 in mice [6], we performed a pentachrome stain on e15.0 and e15.5 embryos. At e15.0, traces of bone (yellow staining) were present in the *GSK-3β* +/+ embryos, while no bone (only blue staining) was appreciated in the *GSK-3β* −/− embryos (**Figure 2** **, first row**). At e15.5, the developing palatine bone in *GSK-3β* +/+ embryos was largely composed of bone (yellow staining), while no bone (only blue staining) was noted in *GSK-3β* −/− embryos (**Figure 2** **, second row**). These data indicate that ossification of the palatine bones is inhibited in *GSK-3β* −/− embryos.

### *GSK-3β* −/− Embryos Exhibit Decreased Osteogenic Gene Expression in the Palatine Bones

**Figure 3:**
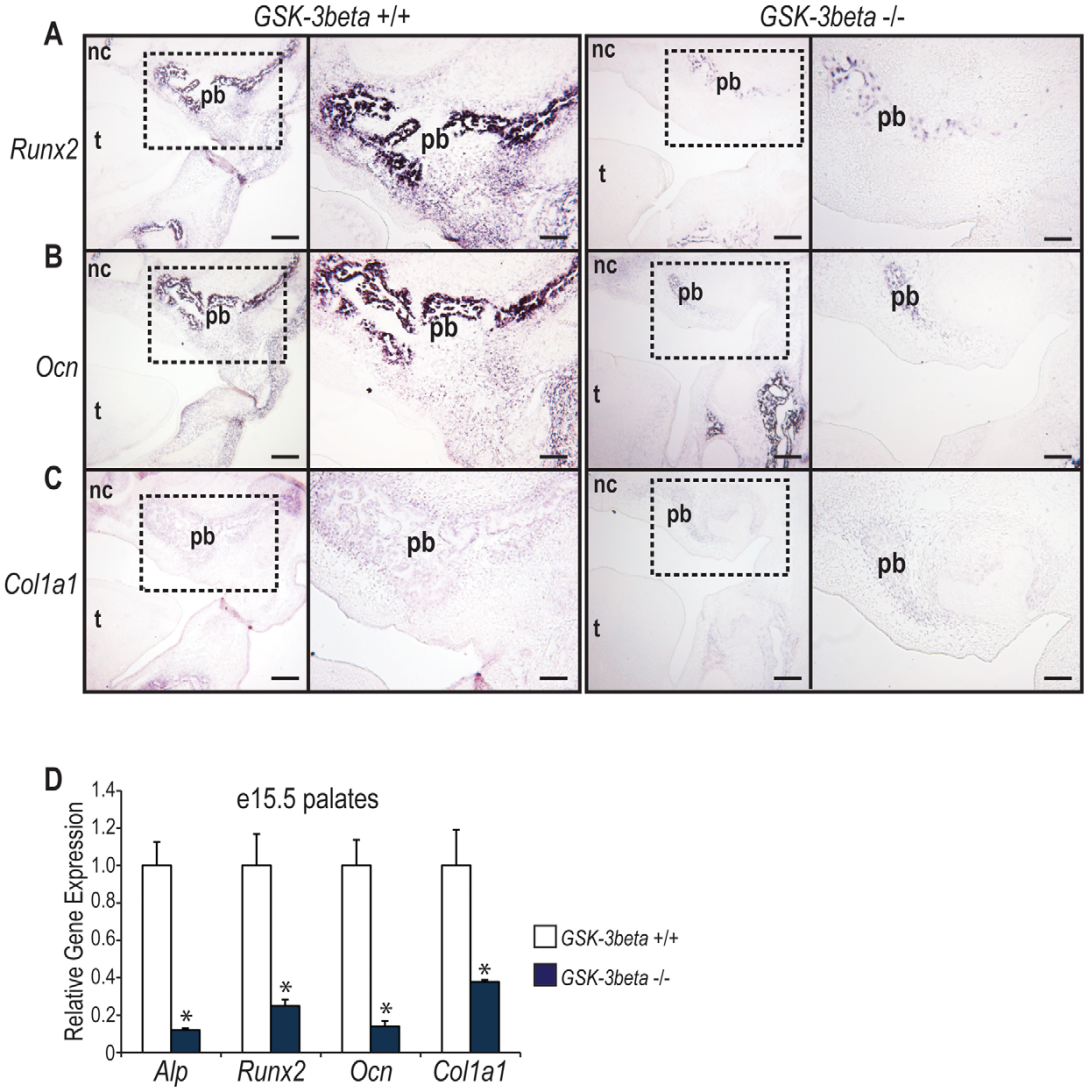
*GSK-3β* −/− embryos exhibit decreased osteogenic gene expression in the palatine bones. (A–C) *In situ* hybridization for the osteogenic genes *Runx2* (A), *Ocn* (B), and *Col1a1* (C). As shown, the intensity and distribution of osteogenic gene transcripts is greater in the *GSK-3β* +/+ embryos than in the *GSK-3β* −/− embryos within and around the palatine bone (pb). The dotted lines in the first and third columns indicate the area shown in higher magnification in the second and fourth columns, respectively. The tongue (t) and nasal cavity (nc) are labeled for orientation purposes. Control experiments using sense probes were performed for all genes (data not shown). Scale bars in the low magnification images (first and third columns) represent 200 µm. Scale bars in higher magnification images (second and fourth) represent 100 µm. (D) qRT-PCR of e15.5 *GSK-3β* +/+ and −/− embryos. *GSK-3β* −/− embryos display significantly decreased levels of the osteogenic genes *Alp*, *Runx2*, *Ocn*, and *Col1a1* by qRT-PCR, in comparison to wild-type littermates. N = 3, *p<0.01.

After confirmation that *GSK-3β* null embryos exhibit decreased ossification of the palatine bones, we next sought to determine whether or not this decrease correlated with a down-regulation in osteogenic gene expression using both *in situ* hybridization and qRT-PCR. In order to determine whether *GSK-3β* null embryos express decreased levels of osteogenic gene markers in the developing palate, we performed an *in situ* hybridization for the specific osteogenic genes *Runt-Related Transcription Factor 2 (Runx2)*, *Osteocalcin (Ocn)*, and *Type 1 Collagen (Col1a1)* on coronal sections of e15.5 *GSK-3β* +/+ and −/− embryos. The embryonic age of e15.5 was chosen as this is the time point at which osteogenic gene markers reach peak expression levels [13]. Additionally, e15.5 is when we begin to appreciate the palatine bone on histology. Not surprisingly, we observed a substantial decrease in both the domain and intensity of signal for *Runx2*, *Ocn*, and *Col1a1* transcripts in the area of the developing palatine bone (**Figure 3A–C**), indicating that *GSK-3β* null embryos exhibit decreased palatal osteogenic gene expression, leading to decreased ossification in the palatine bone and hard palate.

In order to further corroborate our findings from *in situ* hybridization, qRT-PCR was performed on palates dissected from *GSK-3β* +/+ and −/− embryos at e15.5. A significant decrease in the osteogenic gene markers *Alkaline Phosphatase (Alp)*, *Runx2*, *Ocn*, and *Col1a1* was observed by qRT-PCR in *GSK-3β* −/− embryos when compared to their wild-type littermates (**Figure 3D**). Taken together, these data indicate that *GSK-3β* −/− embryos have decreased osteogenic gene expression in the palatine bone of the developing palate, when compared to *GSK-3β* +/+ embryos.

After confirmation that *GSK-3β* null embryos exhibit decreases in osteogenic gene expression, we next sought to determine which signaling pathways were responsible. We assessed both *Wnt* and *Hedgehog* signaling pathways in the *GSK-3β* null embryo as both pathways are regulated by GSK-3β [1] and implicated in craniofacial development [3], [4], [5], in addition to mesenchymal ossification [11], [12], [13].

### *GSK-3β* Null Mutant Embryos Exhibit Increased Wnt Signaling in the Developing Palate

**Figure 4:**
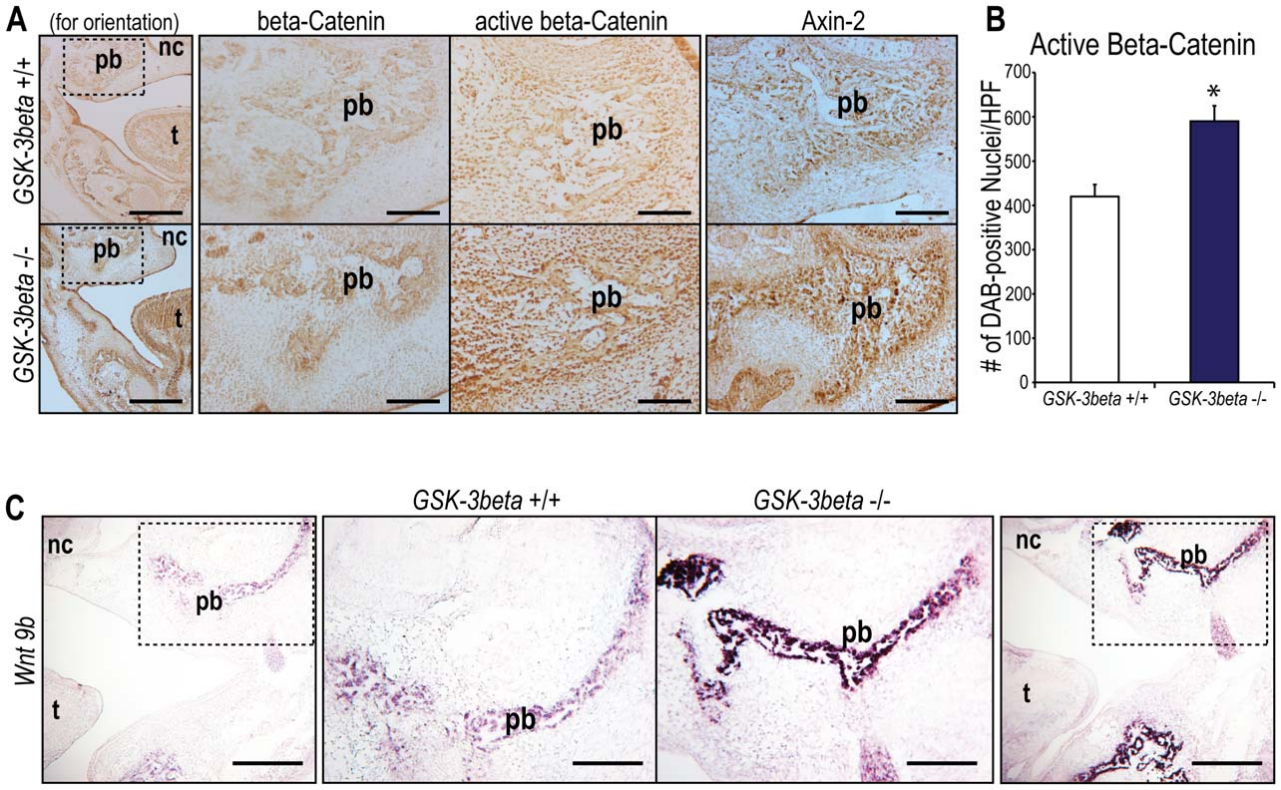
e15.5 *GSK-3β* null embryos exhibit increased canonical *Wnt* signaling in the developing palate. (A) Immunohistochemistry for total β-catenin (second column), active β-catenin (third column), and Axin-2 (fourth column) in *GSK-3β* +/+ and *GSK-3β* −/− embryos. The first column is included for orientation purposes. The dotted lines in the first column indicate the areas shown in higher magnification in the second, third, and fourth columns. As shown, *GSK-3β* −/− embryos have more intense total β-catenin, active β-catenin, and Axin-2 immunostaining than controls in the palatine bone (pb), suggesting that *GSK-3β* −/− embryos have increased canonical *Wnt* signaling compared to *GSK-3β* +/+ embryos. Tongue (t) and nasal cavity (nc) are labeled for orientation purposes. Scale bars in the lower magnification images (first column) represent 200 µm. Scale bars in the higher magnification images (second, third, and fourth columns) represent 100 µm. (B) Quantification of DAB-positive nuclei per high power field, displaying significantly greater active β-catenin positive nuclei in the *GSK-3β* −/− palates when compared to controls. N = 3, *<p0.05. (C) *In situ* hybridization for *Wnt 9b*, a canonical *Wnt* ligand expressed in the craniofacial region. The dotted lines in the first and fourth column indicate the area shown in higher magnification in the second and third columns, respectively. The intensity and distribution of *Wnt9b* transcripts is greater in the *GSK-3β* −/− palates when compared to controls. Tongue (t) and nasal cavity (nc) are labeled for orientation purposes. Scale bars in the lower magnification images (first and fourth column) represent 200 µm. Scale bars in the higher magnification images (second and third columns) represent 100 µm.

We compared canonical *Wnt* signaling between e15.5 *GSK-3β* +/+ and −/− embryos by assessing protein immunoreactivity of both a key mediator of the canonical *Wnt* signaling pathway, β-catenin, and a direct target of the canonical *Wnt* signaling pathway, Axin-2 [15]. When cells are not exposed to a *Wnt* signal, the level of cytosolic β-catenin, in both its active and inactive forms, is kept low through its association with a protein complex, including the protein kinase, GSK-3β. GSK-3β phosphorylates β-catenin, leading to its degradation through the ubiquitin pathway. When cells are exposed to a *Wnt* signal, a conformation change in the GSK-3β protein complex makes GSK-3β unable to phosphorylate β-catenin. The unphosphorylated, active, β-catenin is translocated into the nucleus, which leads to the transcription of downstream *Wnt* target genes [16], [17]. Therefore, cells with increased *Wnt* signaling display increased cytosolic and active β-catenin immunostaining. In addition, we also assessed protein immunoreactivity of a direct target of the canonical *Wnt* signaling pathway, Axin-2 [15]. As shown, *GSK-3β* −/− embryos exhibited a slightly more intense total β-catenin immunostaining compared to controls (**Figure 4A** **, second column**). More importantly, *GSK-3β* −/− embryos displayed increased protein immunostaining of both active β-catenin and Axin-2 in the palatine bone by immunohistochemistry compared to wild-type littermates (**Figure 4A** **, third and fourth columns**). Quantification of DAB-positive nuclei per high power field displayed significantly greater active β-catenin positive nuclei in the *GSK-3β* −/− palates compared to controls (**Figure 4B**). Additionally, several canonical *Wnt* ligand genes are expressed in the craniofacial region, including *Wnt 3* and *Wnt 9b* [18]. Therefore, we performed an *in situ* hybridization for *Wnt 9b* to provide a more complete picture of how deletion of *GSK-3β* impacts *Wnt* signaling. We found that the intensity and distribution of *Wnt9b* transcripts was greater in the *GSK-3β* null embryo compared to wild-type, suggesting that increased *Wnt* signaling results in an upregulation of *Wnt 9b* in the craniofacial region (**Figure 4C**). After confirmation that *GSK-3β* null embryos exhibit increased *Wnt* signaling, we decided to next evaluate *Hedgehog* signaling.

### *GSK-3β* Null Embryos Display Decreased Hedgehog Signaling in the Developing Palate

Both *Wnt* and *Hedgehog* signaling pathways are regulated by GSK-3β [1] and implicated in craniofacial development [3], [4], [5], in addition to mesenchymal ossification [11], [12], [13]. Since *Wnt* signaling activity is enhanced in the *GSK-3β* null embryo, we wanted to determine whether *Hedgehog* signaling was also affected. Therefore, we next investigated whether a difference in *Hedgehog* signaling existed between *GSK-3β* +/+ and −/− embryos.

**Figure 5:**
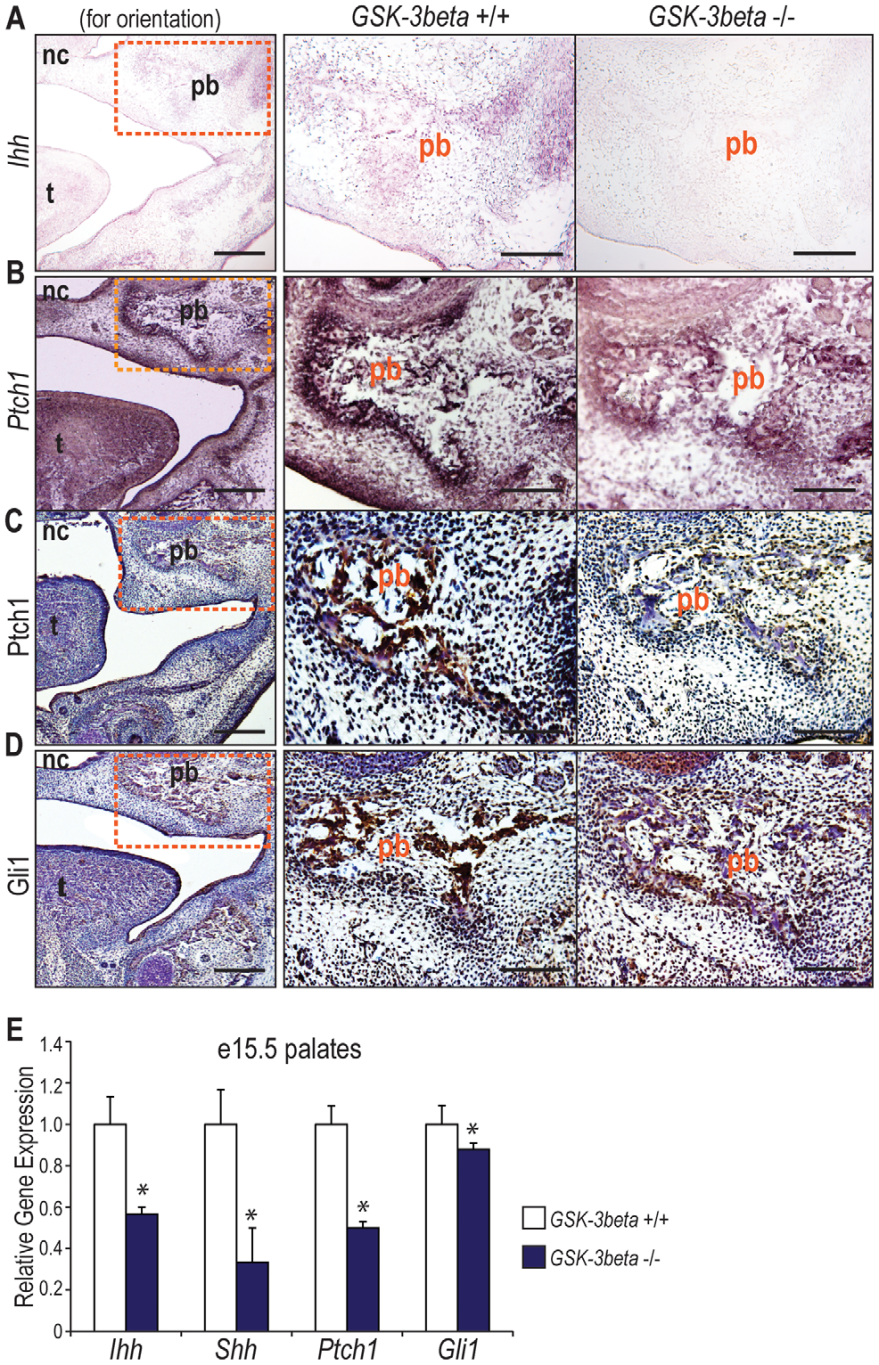
*GSK-3β* null embryos display decreased *Hedgehog* signaling in the developing palate. (A–D) *In situ* hybridization and/or immunohistochemistry of e15.5 coronal sections from *GSK-3β* +/+ and *GSK-3β* −/− embryos to evaluate *in vivo Hedgehog* signaling. For *in situ* hybridizations, the signal appears purple. For immunohistochemistry, the signal is developed with DAB (brown color) and counterstained in hematoxylin (blue). The first column is for orientation purposes. The orange box in the images in the first column represent the area shown in high magnification in the second and third columns. (A) *In situ* hybridrization for the *Hedgehog* ligand, *Ihh*, demonstrating that *GSK-3β* −/− embryos have decreased *Ihh* transcripts in the palatine bone when compared to controls. (B) *In situ* hybridization for the *Hedgehog* receptor, *Ptch1*, demonstrating that *GSK-3β* −/− palates have decreased *Ptch1* transcript in the palatine bone when compared to controls. (C) Immunohistochemistry for the *Hedgehog* receptor, Ptch1, demonstrating that *GSK-3β* −/− palates have decreased Ptch1 immunostaining when compared to controls. (D) Immunohistochemistry of the *Hedgehog* downstream target, Gli1, demonstrating that *GSK-3β* −/− palates have decreased Gli1 protein immunoreactivity in the palatine bone than controls. (E) qRT-PCR of e15.5 *GSK3β* +/+ and −/− palates. The specific genes analyzed include *Hedgehog* ligands, *Ihh* and *Shh*, and the *Hedgehog* downstream target, *Gli1*. *GSK-3β* −/− embryos displayed significantly decreased levels of *Ihh*, *Shh*, and *Gli1*, in comparison to controls. N = 3, *p<0.01.

In order to assess *Hedgehog* signaling *in vivo*, we performed both *in situ* hybridization and immunohistochemistry on e15.5 *GSK-3β* +/+ and −/− embryos for different markers of *Hedgehog* activity (**Figure 5A–D**). This time point was chosen as we have previously shown that *Hedgehog* signaling in the developing palate peaks at e15.5 [13]. An *Ihh* and *Ptch1 in situ* hybridization exhibited decreased transcripts in *GSK-3β* null embryos when compared to their wild-type littermates (**Figure 5A,B**). Additionally, immunohistochemistry was performed, demonstrating that *GSK-3β* null embryos also displayed decreased Ptch1 and Gli1 protein immunoreactivity when compared to controls (**Figure 5C,D**).

In order to further corroborate our findings from *in situ* hybridization and immunohistochemistry, qRT-PCR was performed on e15.5 embryos. *GSK-3β* −/− embryos exhibited a significant decrease in expression of the *Hedgehog* ligands, *Indian Hedgehog (Ihh)* and *Sonic Hedgehog (Shh)*, the *Hedgehog* receptor, *Patched Homolog 1 (Ptch1)*, and the *Hedgehog* downstream target, *GLI-Kruppel family member GLI1 (Gli 1)* by qRT-PCR when compared to controls (**Figure 5E**). Taken together, these data indicate that *Hedgehog* signaling activity is decreased in the palatine bone of *GSK-3β* −/− embryos when compared to their wild-type littermates.

Now that we had confirmed that *GSK-3β* −/− mice exhibit aberrant *Wnt* and *Hedgehog* signaling, we next sought to determine which of these pathways accounted for the impaired palatal ossification found in *GSK-3β* null embryos.

### Upregulation of the Wnt Signaling Pathway Leads to Decreased Osteogenesis *In Vitro*

**Figure 6:**
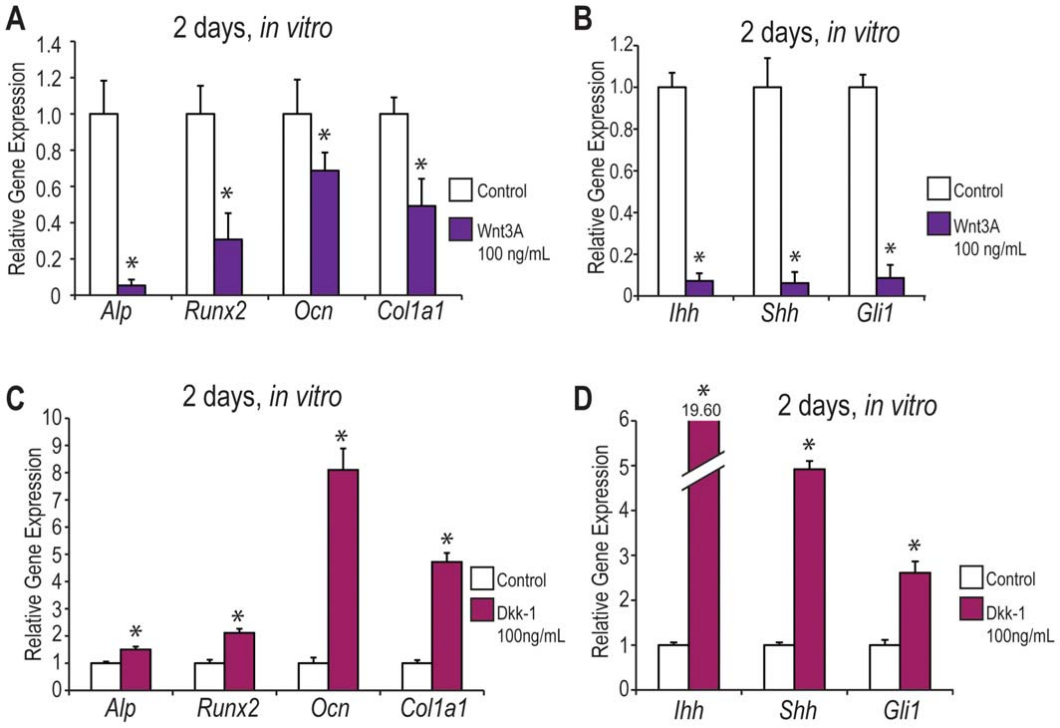
Canonical *Wnt* signaling activation makes wild-type palates more *GSK-3β* “knock-out like” *in vitro*. (A–B) e13.5 wild-type, CD-1 palate cultures were treated with DMEM F12 +/− supplementation with Wnt3A (100 ng/mL) for 2 days. qRT-PCR was performed to evaluate both osteogenic gene expression (A) and *Hedgehog* signaling activity (B). N = 3, *p<0.01. (A) Wild-type palates treated with Wnt3A for 2 days exhibited significantly decreased expression of the osteogenic genes *Alp*, *Runx2*, *Ocn*, and *Col1a1* by qRT-PCR when compared to controls. (B) Palates treated with Wnt3A for 2 days displayed decreased *Hedgehog* signaling activity, with significantly decreased levels of *Ihh*, *Shh*, and *Gli1* by qRT-PCR. (C–D) e13.5 wild-type, CD-1 palate cultures were treated with DMEM F12 +/− supplementation with Dkk-1 (100 ng/mL) for 2 days. qRT-PCR was performed to evaluate both osteogenic gene expression (C) and *Hedgehog* signaling activity (D). N = 3, *p<0.01. (C) Wild-type, CD-1 palates treated for 2 days with Dkk-1 demonstrated a significant increase in the osteogenic genes *Alp*, *Runx2*, *Ocn*, and *Col1a1*. (D) Palates treated for 2 days with Dkk-1 demonstrated significant increases in the *Hedgehog* ligands, *Ihh* and *Shh* and the downstream target, *Gli1*.

In order to determine whether enhanced canonical *Wnt* signaling alone could lead to a decrease in palatal osteogenesis, wild-type, CD-1 palate cultures were established at e13.5 and treated with DMEM F12 or DMEM F12 supplemented with Wnt3A (100 ng/mL). This time point was chosen as previous studies have demonstrated that *GSK-3β* −/− palates can be “rescued” *in vivo* by restoring endogenous *GSK-3β* levels between e13.5 and e15.0 [1]. Viability of cells after 4 days in culture using this palate culture technique has previously been demonstrated with IVIS technology [13]. After palate cultures were established, qRT-PCR was performed after 2 days to investigate both osteogenic gene expression and *Hedgehog* signaling in Wnt3A-treated palate cultures (**Figure 6A–B**). After 2 days in culture, a significant decrease was noted in the ostegenic gene markers *Alp*, *Runx2*, *Ocn*, and *Col1a1* in Wnt3A-treated palates when compared to controls (**Figure 6A**). Since Wnt3A treatment alone led to a significant decrease in palatal osteogenesis, we wanted to determine whether increased *Wnt* signaling also affected the *Hedgehog* signaling pathway.

### Upregulation of the Wnt Signaling Pathway Leads to Decreased Hedgehog Signaling Activity *In Vitro*

Palate cultures treated with Wnt3A led to a significant decrease in *Hedgehog* ligands, *Ihh* and *Shh*, and the downstream target, *Gli1* by qRT-PCR after 2 days in culture (**Figure 6B**). These data confirm that an increase in canonical *Wnt* signaling results in decreases of both osteogenic gene markers and *Hedgehog* signaling activity and is therefore sufficient to mimic the gene expression pattern found in *GSK-3β* null embryo palates.

### Inhibition of the Wnt Signaling Pathway Leads to Increased Osteogenesis *In Vitro*

Because treatment with Wnt3A led to decreased osteogenesis *in vitro*, we wanted to determine whether inhibition of canonical *Wnt* signaling would lead to increases in osteogenesis in the developing palate. Therefore, wild-type, CD-1 palate cultures at e13.5 were treated with DMEM F12 +/− supplementation with a canonical *Wnt* inhibitor, Dickkopf-related protein 1 (Dkk-1) at 100 ng/mL for 2 days (**Figure 6C**). After 2 days in culture, palates treated with Dkk-1 displayed significant increases in the osteogenic genes *Alp*, *Runx2*, *Ocn*, and *Col1a1* by qRT-PCR (**Figure 6C**).

### Inhibition of Wnt Signaling Leads to Increased Hedgehog Signaling

Because treatment with Wnt3A led to decreased *Hedgehog* signaling *in vitro*, we also wanted to determine whether the inhibition of *Wnt* signaling would lead to increased *Hedgehog* signaling. e13.5 wild-type, CD-1 palate cultures were treated with DMEM F12 +/− supplementation with Dkk-1 (100 ng/mL) for 2 days (**Figure 6D**). Wild-type palates treated with the canonical *Wnt* inhibitor demonstrated significantly increased levels of *Hedgehog* ligands, *Ihh* and *Shh*, and the downstream target, *Gli1* (**Figure 6D**).

### Inhibition of Hedgehog Signaling Also Leads to Decreased Ostegenesis *In vitro*

**Figure 7:**
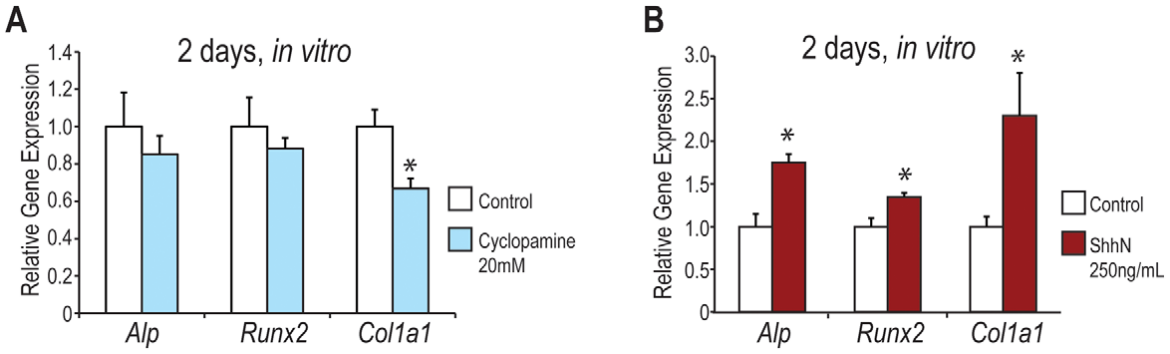
Inhibition of the *Hedgehog* pathway leads to decreased ostegenesis *in vitro*. (A–B) qRT-PCR of e13.5 wild-type, CD-1 palate cultures treated with DMEM F12 +/− supplementation with cyclopamine (20 mM) or Shh-N (250 ng/mL) for 2 days. N = 3, *p<0.01. (A) Wild-type, CD-1 palates treated with cyclopamine for 2 days exhibited a decrease in osteogenic markers *Alp*, *Runx2*, and *Col1a1* by qRT-PCR, with a significant decrease in *Col1a1*. (B) Wild-type palates treated with Shh-N exhibited significant increases in the osteogenic genes *Alp*, *Runx2*, and *Col1a1*.

Because palate cultures treated with Wnt3A led to both a decrease in osteogenic gene markers in addition to a decrease in *Hedgehog* signaling, we next wanted to determine whether the inhibition of *Hedgehog* signaling with cyclopamine would also lead to a decrease in palatal osteogenesis. Previous studies with *Ihh* null embryos suggested that inhibition of *Hedgehog* signaling alone results in significant decreases of osteogenic gene expression in the developing palate [13]. Wild-type, CD-1 palate cultures were established at e13.5 and treated with DMEM F12 +/− supplementation with cyclopamine (20 mM) (**Figure 7A**). As expected, wild-type palate cultures treated with cyclopamine for 2 days exhibited a decrease in the osteogenic gene markers *Alp*, *Runx2*, and *Col1a1* by qRT-PCR, with a significant decrease in *Col1a1* when compared to controls (**Figure 7A**).

### Upregulation of Hedgehog Signaling Activity in the Developing Palate Results in Increased Osteogenesis *In Vitro*

Because inhibition of the *Hedgehog* signaling pathway with cyclopamine resulted in decreased palatal osteogenesis, we wanted to determine whether the converse was also true. Therefore, e13.5 wild-type, CD-1 palate cultures were treated with DMEM F12 +/− supplementation with a recombinant *Hedgehog* ligand, Shh-N (250 ng/mL) for 2 days (**Figure 7B**). After 2 days in culture, palates treated with Shh-N displayed significant increases in osteogenic gene expression (*Alp*, *Runx2*, and *Col1a1*) by qRT-PCR (**Figure 7B**).

### Alterations in Hedgehog Signaling Results in No Obvious Changes in Canonical Wnt Signaling

**Figure 8:**
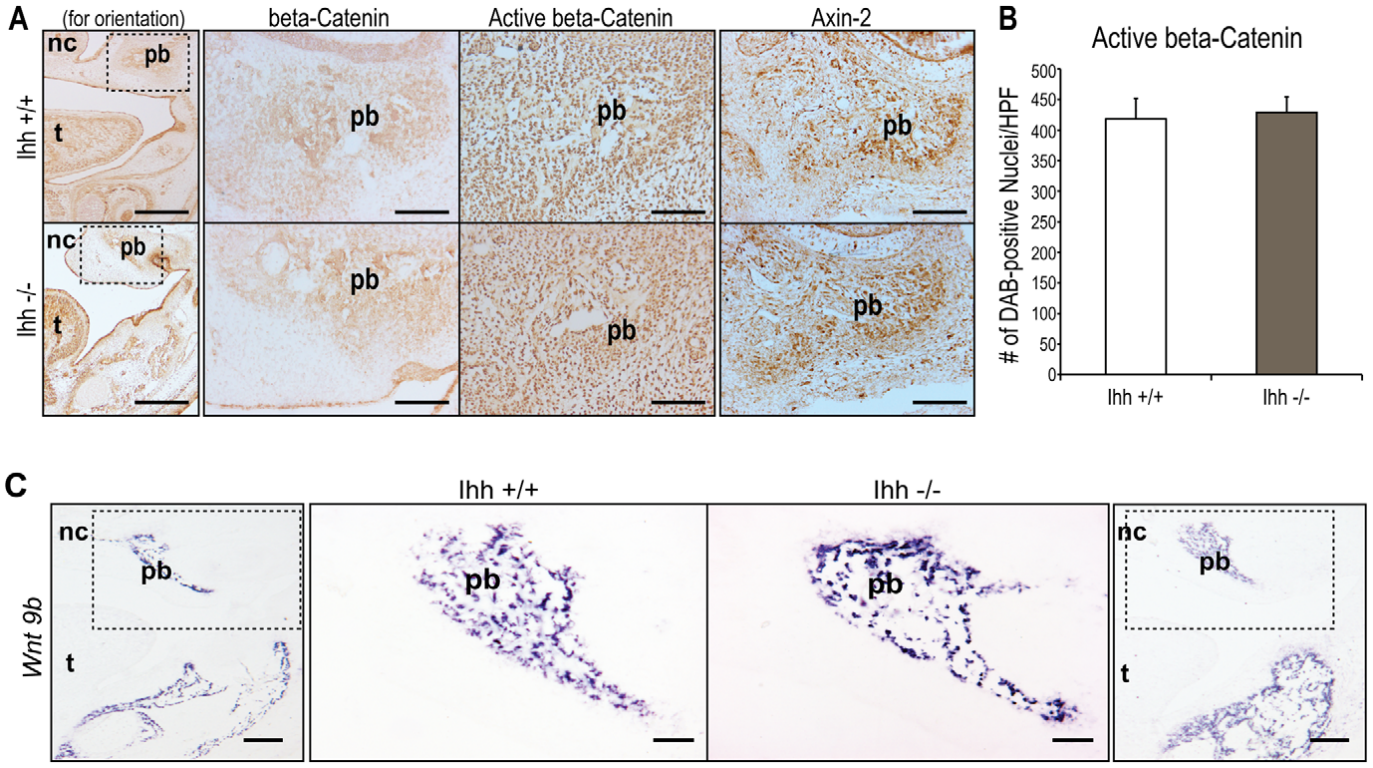
Canonical *Wnt* signaling is similar in both *Ihh* +/+ and *Ihh* −/− embryos. (A) Immunohistochemistry for β-catenin, active β-catenin, and Axin-2 in e15.5 coronal sections from *Ihh* +/+ and *Ihh* −/− embryos. Little to no difference is appreciated between *Ihh* +/+ and *Ihh* −/− embryos. For orientation, the first column shows a low magnification image with dotted lines around the area shown in high magnification in the second, third, and fourth columns. Tongue (t) and nasal cavity (nc) are labeled for orientation purposes. Scale bars in the lower magnification images (first column) represent 200 µm. Scale bars in the higher magnification images (second, third, and fourth columns) represent 100 µm. (B) Quantification of DAB-positive nuclei per high power field demonstrating no significant differences in active β-catenin immunostaining between *Ihh* +/+ and −/− palates. N = 3, *p<0.05. (C) *In situ* hybridization of *Wnt 9b*, a canonical *Wnt* ligand expressed in the craniofacial region. Similarly, no difference in the domain or intensity of *Wnt9b* transcripts is noted between groups. Scale bars in the lower magnification images (first and fourth column) represent 200 µm. Scale bars in the higher magnification images (second and third columns) represent 100 µm.

We performed immunohistochemistry and *in situ* hybridization on coronal sections of e15.5 *Ihh* null embryos and their wild-type littermate controls in order to evaluate canonical *Wnt* signaling activity (**Figure 8**). No differences were noted in protein staining of either total or active ß-catenin, or Axin-2, a direct target of the canonical *Wnt* signaling pathway (**Figure 8A**). In addition, quantification of DAB-positive nuclei per high power field displayed no significant differences in active β-catenin immunostaining between *Ihh* +/+ and −/− palates (**Figure 8B**). Similarly, no differences were noted in the canonical *Wnt* ligand, *Wnt 9b*, which is normally expressed in the craniofacial region [18] (**Figure 8C**). While alterations in canonical *Wnt* signaling affected *Hedgehog* signaling, alterations in the *Hedgehog* pathway did not appear to affect the *Wnt* pathway. These data suggest that canonical *Wnt* signaling is upstream of the *Hedgehog* pathway during secondary palate development.

### Wnt inhibition “rescues” *GSK-3β* null embryo palates

**Figure 9:**
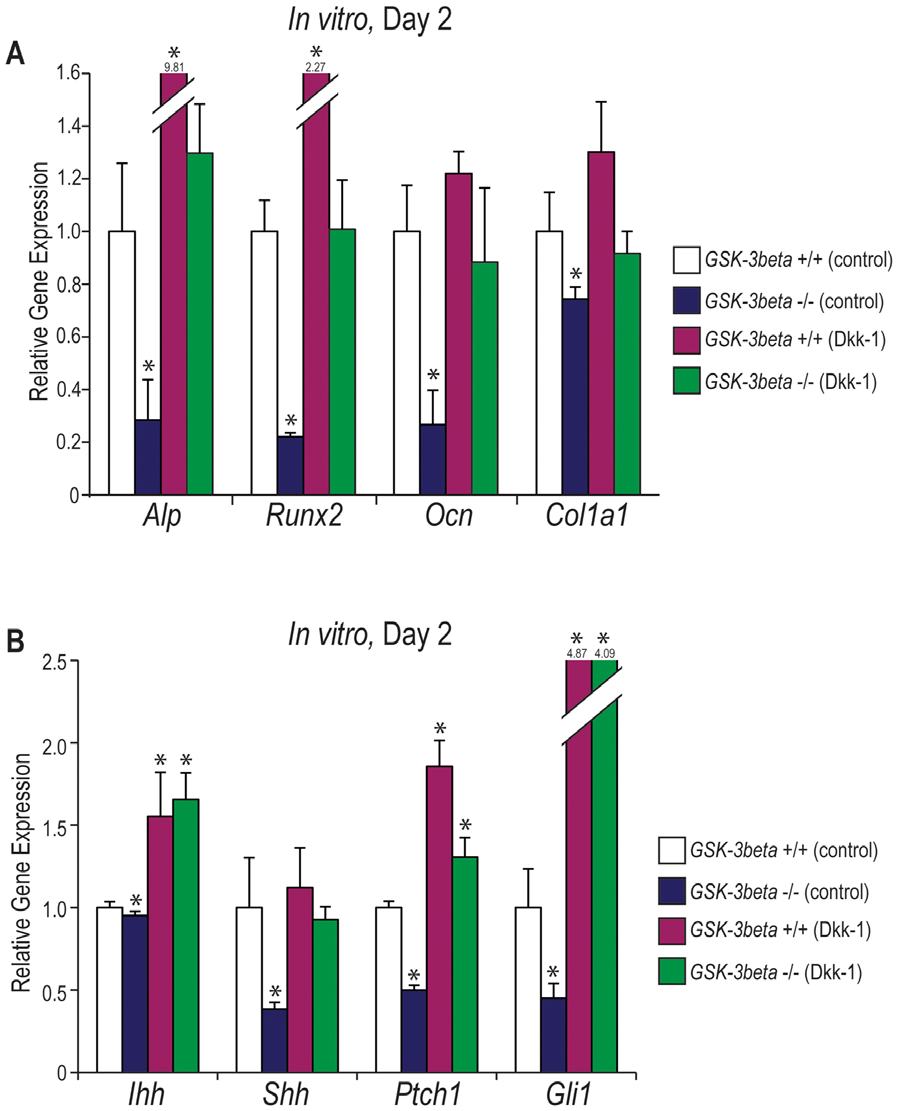
*Wnt* signaling inhibition “rescues” *GSK-3β* null embryo osteogenic gene expression and *Hedgehog* signaling activity. (A–B) e13.5 *GSK-3β* +/+ and −/− embryo palate cultures treated for 2 days with DMEM F12 +/− supplementation with Dkk-1 (100 ng/mL). (A) qRT-PCR for the osteogenic gene markers *Alp*, *Runx2*, *Ocn*, and *Col1a1*. N = 3, *<0.01. (B) qRT-PCR for members of the *Hedgehog* signaling pathway (*Ihh*, *Shh*, *Ptch1*, and *Gli1*). N = 3, *<0.01.

If canonical *Wnt* signaling is truly upstream of the *Hedgehog* pathway, treating *GSK-3β* null embryo palate cultures with Dkk-1, a *Wnt* inhibitor, should “rescue” both osteogenic gene expression and *Hedgehog* signaling activity in the mutant embryo. Therefore, e13.5 palate cultures were again established. This time, however, *GSK-3β* null embryos, in addition to their wild-type littermates, were treated with DMEM F12 +/− supplementation with Dkk-1 (100 ng/mL) for 2 days (**Figure 9**). We found that *GSK-3β* null embryos continued to express significantly lower levels of osteogenic gene markers (*Alp*, *Runx2*, *Ocn*, and *Col1a1*) by qRT-PCR, when compared to their wild-type littermates (**Figure 9A** **, white and blue bars**). Interestingly, however, osteogenic gene expression (*Alp*, *Runx2*, *Ocn*, and *Col1a1*) in the *GSK-3β* null embryos treated with Dkk-1 resulted in non-significant differences, when compared to their wild-type littermates treated with DMEM F12 alone (**Figure 9A** **, white and green bars**). Additionally, similar results were observed after performing qRT-PCR for members of the *Hedgehog* signaling pathway (**Figure 9B**). While *GSK-3β* null embryos treated with DMEM F12 alone expressed significantly lower levels of the *Hedgehog* ligands, *Ihh* and *Shh*, receptor, *Ptch1*, and downstream target, *Gli 1*, when compared to wild-type littermates under the same conditions (**Figure 9B** **, white and blue bars**), following treatment with Dkk-1, *GSK-3β* null embryos expressed significantly higher levels of *Ihh*, *Ptch1*, and *Gli 1*, when compared to wild-type littermates treated with DMEM F12 alone (**Figure 9B** **, white and green bars**). No significant differences were noted in expression levels of *Shh* between *GSK-3β* +/+ embryos treated with DMEM F12 alone and *GSK-3β* −/− embryos treated with Dkk-1 (**Figure 9B** **, white and green bars**). These data serve to further support our hypotheses that *GSK-3β* null embryos display inhibited ossification of the secondary palate, as a result of both *Wnt* signaling activation and *Hedgehog* signaling inhibition, and additionally, that *Wnt* signaling is upstream of *Hedgehog* signaling during secondary palate development.

## Discussion

In this study, we provide evidence for enhanced canonical *Wnt* signaling in the *GSK-3β* null embryo in the region of the palatine bone with simultaneous decreases in *Hedgehog* signaling. Importantly, our study demonstrates that inhibition of *GSK-3ß* leads to a decrease in the osteogenic differentiation of palatal mesenchymal cells, leading to the underdevelopment of the palatine bones. Additionally, through the use of palate cultures, we found that we can mimic the changes we observed in the *GSK-3ß* null palatine bone in both osteogenic gene expression and *Hedgehog* signaling activity through treatment with recombinant Wnt3A alone. Furthermore, it appears that canonical *Wnt* signaling may be upstream of *Hedgehog* signaling during secondary palate development as increases or decreases in *Wnt* signaling resulted in decreased or increased *Hedgehog* signaling activity, respectively, while alterations in *Hedgehog* signaling did not appear to affect canonical *Wnt* signaling. Lastly, we found that *Wnt* signaling inhibition in *GSK-3β* null embryos was sufficient to “rescue” both osteogenic gene expression and *Hedgehog* signaling in the mutant embryo. Taken together, these data describe a palatal phenotype and provide a possible mechanism behind the observation that *GSK-3ß* is important for palatine bone ossification.

Several *Wnt* antagonists have been described including Dickkopfs (DKKs) and Sclerostin [19], [20], [21]. Dkk-1 specifically suppresses the canonical *Wnt* pathway by binding to the *Wnt* receptor, LRP5/6 [22]. Therefore, Dkk-1 acts upstream from GSK-3*ß* to inhibit canonical *Wnt* signaling. However, it remains possible to inhibit *ß*-catenin-dependent *Wnt* signaling in both wild-type and GSK-3*ß* null embryos. This is because two functionally redundant isoforms of GSK-3 exist in mammals, GSK-3α and GSK-3*ß* [23]. This functional redundancy allows GSK-3*ß* null embryos to exhibit some GSK-3 inhibition of *ß* –catenin, but not enough to fully compensate for the loss of GSK-3*ß* [23]. For this reason, we were able to inhibit canonical *Wnt* signaling in both wild-type and GSK-3*ß* null embryos.

Analysis of the canonical *Wnt* signaling pathway in the developing palate has revealed that there is baseline activation in wild-type mice. However, with deletion of the *GSK-3ß* gene, we noticed a significant upregulation in canonical *Wnt* signaling. Our observation that Dkk-1 stimulates an increase in osteogenic gene expression in palate cultures leads us to believe that the precise regulation of canonical *Wnt* signaling likely plays a role in palatal mesenchyme osteogenic differentiation.

If the impaired palatal ossification seen in our *GSK-3ß* null embryo is due, in part, to sustained activation of the canonical *Wnt* pathway, which subsequently leads to inhibition of the *Hedgehog* signaling pathway, we would expect that Wnt-3A treated wild-type palate cultures would lead to similar decreases in osteogenic differentiation and *Hedgehog* signaling activity as observed in *GSK-3β* −/−palates. Conversely, supplementation with the canonical *Wnt* inhibitor, Dkk-1, should enhance osteogenic gene expression and *Hedgehog* signaling activity. In our organ culture assay, we found these results indeed to be the case. Taken together, these data confirm that the impaired ossification of the secondary palate is due, at least in part, to enhanced canonical *Wnt* signaling in *GSK-3ß* null embryos.

Similarly, if decreases in the *Hedgehog* pathway alone also contribute, in part, to the impaired palatal ossification observed in *GSK-3ß* null embryos, we would expect palate cultures treated with the *Hedgehog* inhibitor, cyclopamine, to display decreases in osteogenic gene expression. Conversely, palate cultures treated with the *Hedgehog* agonist, Shh-N, should result in increases in osteogenic gene expression. We found this again to be the case, confirming that the decreased palatal mesenchymal osteogenesis observed in the *GSK-3ß* null embryo is also due, in part, to decreases in *Hedgehog* signaling.

The increase in canonical *Wnt* signaling that coincides with a decrease in *Hedgehog* signaling observed in *GSK-3β* null embryos suggests that there may be a coordination between these two pathways in palatal mesenchymal cell osteogenesis. Previous studies have shown that increases in canonical *Wnt* [12] or decreases in *Hedgehog* signaling [11] result in inhibited ossification. In light of these previous studies, our results suggest that increases in *Wnt* signaling, such as those observed in the *GSK-3ß* null embryo, act to inhibit the ossification program already in place. Furthermore, the inhibition of *Hedgehog* signaling likely serves to only further inhibit palatal ossification. Therefore, while aberrations in either the *Wnt* and *Hedgehog* pathway alone could explain the impaired palatal ossification found in *GSK-3ß* null embryos, it seems likely that both increased *Wnt* signaling and decreased *Hedgehog* signaling contribute to the palatal phenotype observed in these mutant embryos.

Interestingly, a recent study by Nalesso et al. suggests that the non-canonical *Wnt* signaling pathway may also play a role in the palatal ossification process [24]. While Wnt-3A was previously believed to specifically upregulate the canonical *Wnt* pathway alone [25], [26], Nalesso et al. found that treatment of articular chondrocytes with Wnt-3A led to an upregulation of both canonical, *ß*-catenin-dependent, and non-canonical, CaMKII-dependent, signaling. Additionally, they found these two pathways to be reciprocally inhibitory [24]. While their study was performed to evaluate chondrogenesis of articular chondrocytes rather than osteogenesis of palatal mesenchyme, future studies on how the non-canonical, CaMKII-dependent pathway affects the palatal ossification process may shed further light on our findings.

In summary, based on our findings, we propose that *GSK-3ß* inhibition leads to canonical *Wnt* signaling activation and subsequent decreases in *Hedgehog* signaling. Both the enhanced canonical *Wnt* signaling and decreased *Hedgehog* signaling contribute to the impaired palatal osteogenesis observed in the *GSK-3ß* null embryos. In addition, because alterations in the canonical *Wnt* pathway led to aberrant *Hedgehog* signaling, while alterations in *Hedgehog* signaling led to no obvious changes in the canonical *Wnt* pathway, it seems likely that canonical *Wnt* functions upstream of *Hedgehog* signaling during secondary palate development. Moreover, the fact that we were able to “rescue” both osteogenic gene expression and *Hedgehog* signaling activity in the GSK-3β −/− palates with a *Wnt* inhibitor provides even stronger evidence for the above stated hypotheses.

Craniofacial clefts, and specifically, clefting of the secondary osseous palate remain a significant biomedical burden. Therefore, understanding the pathways behind secondary palate formation may allow us to better develop therapeutic modalities to treat these patients.

## Materials and Methods

### Ethics Statement

All efforts were made to ameliorate suffering of animals. Animals were housed in the Research Animal Facility on Stanford University campus. The facility, accessed by authorized personnel only, is temperature, ventilation and illumination controlled. Mice have access to feed and water *ad libitum*. All mice housing conforms to NIH *Guide* standards, the Animal Welfare Act, and ILAR *guide*. Transportation of animals was performed based on the “Guidelines for Transportation of Animals from the Stanford Centralized Animal Facilities,” developed by the Administrative Panel on Laboratory Animal Care (A-PLAC). All animal procedures were approved by Stanford A-PLAC, Protocol #9999. No cell lines were used.

### Animals

The transgenic mouse lines for *GSK-3ß* were a gift from J. Woodgett and have been described previously [1], [27]. All of the experiments shown on *GSK-3β* −/− embryos were performed in outbred CD-1 mice; however, we found the same cleft palate phenotype in the original *GSK-3ß* −/− mice. *Ihh* +/− mice were mated to generate *Ihh* −/− embryos on a C57B6 background. *Ihh* +/− mice were mated to obtain both *Ihh* +/+ and *Ihh* −/− mice [11]. For all experiments, animals were bred over night and the day of vaginal plug was considered to be e0.5 days of gestation.

### Palatal shelf dissection

Wild-type, *GSK-3ß* +/+ and *GSK-3ß*−/− embryos were harvested at e13.5 of gestation and immediately placed on ice in cold, sterile, phosphate-buffered saline. Microdissection was performed on the individual embryos to isolate and remove the palatal shelves. Palatal shelves were transferred onto 0.4-µm pore-size filter inserts (Falcon cell culture insert; Becton Dickinson, Franklin Lakes, N.J.) and placed in organ culture plates (Corning Inc., Corning, N.Y.). *In vivo* orientation was maintained throughout. Palatal shelves were cultured with the paired shelves in parallel with the medial edge epithelium of each palate facing each other with the nasal side down onto the filter inserts. Filter inserts were suspended on top of wells containing Dulbecco minimal essential medium/Ham's F12 growth medium supplemented with 300 µg/ml l-glutamate, 50 µg/ml glycine, 100 µg/ml ascorbate, 1% penicillin/streptomycin (all from Gibco, Grand Island, N.Y.) at 37°C in a 5% CO2 environment [28]. Palate cultures were then treated with the above +/− mouse recombinant Sonic Hedgehog/Shh, N terminus (250 ng/mL), recombinant mouse Wnt3A (100 ng/mL), recombinant mouse Dkk-1 (100 ng/mL), or Cyclopamine (20 mM) (R&D systems, Minneapolis, MN). Specimens were examined microscopically 4 hours after initial placement of the paired palatal shelves into culture, to determine whether any premature migration or change in orientation of shelves had occurred requiring readjustment to the originally recorded separation distance and alignment. Palates were harvested after 48 hours and RNA was isolated immediately. In addition, e15.5 *GSK-3β* +/+ and −/− palates were dissected and immediately snap frozen for later RNA isolation.

### RNA Isolation and Real-Time PCR

Once removed from culture, palates were pooled (N = 3 distinct pooled samples, consisting of 2 microdissected palates per sample), and homogenized by sonification. RNA was isolated per the manufacturer's instructions (RNeasy Kit, Qiagen Sciences, Maryland), genomic DNA was removed (DNA-free kit, Ambion, Austin, TX), and a total of 1 ug RNA was reverse-transcribed (Taqman Reverse Transcription Reagents, Applied Biosystems, Foster City, CA). Quantitative real-time PCR was carried out using the Applied Biosystems Prism 7900HT Sequence Detection System and Power Sybr Green Mastermix (Applied Biosystems, Foster City, CA). Specific primers were designed based on PrimerBank sequences (http://pga.mgh.harvard.edu/primerbank/). Sequences are shown in **Table 1**. All reactions were performed in triplicate; relative values were calculated to housekeeping gene (*GAPDH*) and presented as means and standard deviations.

**Table 1:** Quantitative PCR Genes and Primer Sequences.

*Gene Name*	*Forward primer sequence (5′ to 3′)*	*Reverse primer sequence (5′ to 3′)*
*Alkaline Phosphatase*	GTTGCCAAGCTGGGAAGAACAC	CCCACCCCGCTATTCCAAAC
*Gapdh*	AGGAGTATATGCCCGACGTG	TCGTCCACATCCACACTGTT
*Gli1*	TCGACCTGCAAACCGTAATCC	TCCTAAAGAAGGGCTCATGGTA
*Ihh*	GCTTCGACTGGGTGTATTACG	GCTCGCGGTCCAGGAAAAT
*Ptc1*	GCCAAGCCCTAAAAAAAT	ACCACAATCAATCTCCTG
*Runx2*	CGGTCTCCTTCCAGGATGGT	GCTTCCGTCAGCGTCAACA
*Shh*	AAAGCTGACCCTTTAGCCTA	TTCGGAGTTTCTTGTGATCTTCC
*Type I Collagen*	AACCCGAGGTATGCTTGATCT	CCAGTTCTTCATTGCATTGC

### Tissue Processing and Histology

For histological analysis, sections were prepared from tissues fixed overnight in 0.4% PFA, decalcified in 19% EDTA (pH 7.4) at 4°C and dehydrated through graded ethanol for paraffin embedding. For histological staining, 5 µm paraffin-embedded tissue sections were stained with Movat's Pentachrome bone stain. Immunohistochemistry was performed on select slides for β-catenin, Ptch1, and Gli1 (Santa Cruz Laboratories, Santa Cruz, CA), in addition to active β-catenin (Millipore, Billerica, MA) and Axin 2 (Abcam, Cambridge, MA). Slides were deparaffinized and rehydrated. Endogenous peroxidase activity was quenched with 3% hydrogen peroxide in methanol. Antibodies used included rabbit polyclonal anti-β-catenin, (1∶50 in dilution, Santa Cruz Laboratories, Santa Cruz, CA), rabbit polyclonal anti-Ptch1 (1∶50 in dilution, Santa Cruz Laboratories, Santa Cruz, CA), rabbit polyclonal anti-Gli1 (1∶80 in dilution, Santa Cruz Laboratories, Santa Cruz, CA), mouse monoclonal anti-active β-catenin (1∶50 in dilution, Millipore, Billerica, MA), and rabbit polyclonal anti-Axin-2 (1∶400 dilution, Abcam, Cambridge, MA). Rabbit polyclonal antibodies were blocked in 5% goat serum (Vector Laboratories, Burlingame, CA); mouse monoclonal antibody was blocked in 5% horse serum (Vector Laboratories, Burlingame, CA). Appropriate biotinylated secondary antibodies were used in 1∶1000 dilution (Vector Laboratories, Burlingame, CA). The Vectastain ABC system (Vector Laboratories, Burlingame, CA) was used according to the manufacturer's instructions. Visualization was with diaminobenzidine solution (Zymed Laboratories, South San Francisco, CA). Slides without primary antibody were used as a negative control. No less than 5 slides were stained for each antibody per animal (N = 3).

Quantification of active β-catenin positive nuclei amongst *GSK-3β* and *Ihh* +/+ and −/− embryos was performed using the Count Tool on Adobe Photoshop. Photographs of active β-catenin immunostaining on coronoal sections were taken at 40× magnification (3 animals per group, 5 slides per animal). The number of brown stained nuclei were counted by a single blinded investigator, and confirmed by a second, independent investigator.

*In situ* hybridization was performed on select slides for mouse *Runx2*, *Ocn*, *Col1a1*, *Ihh*, and *Ptc1* as previously described [29]. Briefly, the template was amplified from embryonic mouse cDNA by PCR using sequence-specific primers that included either the T7 or T3 RNA promoter region to make both anti-sense and sense probes, respectively. Anti-sense and sense riboprobe was transcribed with T7 RNA polymerase and T3 RNA polymerase, respectively, in the presence of Dig-11-UTP (Roche). Sections were incubated at 63°C for 12 hrs in hybridization buffer (Ambion, Austin TX) containing riboprobe at ∼1 ug/mL probe. Slides were blocked with 10% lamb serum (Invitrogen, Carlsbad, CA), 1% Boehringer-Mannheim Blocking Reagent (Roche), and levamisole, and developed using NBT and BCIP for color. Non-specific binding was minimized by high stringency hybridization conditions. For all assays, sense probes were used side-by-side with minimal background.

### Whole mount skull preparation

To evaluate the palate phenotype of wild-type, *GSK-3ß*−/+ and *GSK-3ß*−/− embryos, whole mount bone and cartilage staining was performed as previously described [29]. Specimens were fixed in 100% ethanol for 48 h, transferred to acetone for 48 h, stained in 0.15% alcian blue in 20% glacial acetic/80% ethanol for 24 h, fixed in ethanol for 24 h and cleared in 1% aqueous KOH for 24–31 h prior to staining in 0.005% alizarin red in 1% KOH for 15 h. The tissue was cleared through 20% glycerol 1% KOH for 24–72 h, followed by increasing concentrations of glycerol in dH_2_O up to 70%.

Palatine bone area was quantified using the Lasso Tool on Adobe Photoshop. Photographs were taken of the palatine bones at 4× magnification. The absolute number of pixels was quantified using Adobe Photoshop. N = 3 animals per group.

### Statistical analyses

Means and standard deviations were calculated from all numerical data. In graphs, all bars represent means whereas all error bars represent one standard deviation. Statistical analyses were performed using the Welch's two-tailed t-test when comparing two groups with unequal standard deviations. *P<0.01 was considered significant, unless otherwise stated.
